# Complete In Vitro Life Cycle of *Trypanosoma congolense*: Development of Genetic Tools

**DOI:** 10.1371/journal.pntd.0000618

**Published:** 2010-03-02

**Authors:** Virginie Coustou, Fabien Guegan, Nicolas Plazolles, Théo Baltz

**Affiliations:** Laboratoire de Microbiologie Cellulaire et Moléculaire et Pathogénicité, UMR-5234 CNRS, Université Victor Segalen Bordeaux 2, Bordeaux, France; Instituto Oswaldo Cruz, Fiocruz, Brazil

## Abstract

**Background:**

Animal African trypanosomosis, a disease mainly caused by the protozoan parasite *Trypanosoma congolense*, is a major constraint to livestock productivity and has a significant impact in the developing countries of Africa. RNA interference (RNAi) has been used to study gene function and identify drug and vaccine targets in a variety of organisms including trypanosomes. However, trypanosome RNAi studies have mainly been conducted in *T. brucei*, as a model for human infection, largely ignoring livestock parasites of economical importance such as *T. congolense*, which displays different pathogenesis profiles. The whole *T. congolense* life cycle can be completed *in vitro*, but this attractive model displayed important limitations: (i) genetic tools were currently limited to insect forms and production of modified infectious BSF through differentiation was never achieved, (ii) *in vitro* differentiation techniques lasted several months, (iii) absence of long-term bloodstream forms (BSF) *in vitro* culture prevented genomic analyses.

**Methodology/Principal Findings:**

We optimized culture conditions for each developmental stage and secured the differentiation steps. Specifically, we devised a medium adapted for the strenuous development of stable long-term BSF culture. Using Amaxa nucleofection technology, we greatly improved the transfection rate of the insect form and designed an inducible transgene expression system using the IL3000 reference strain. We tested it by expression of reporter genes and through RNAi. Subsequently, we achieved the complete *in vitro* life cycle with dramatically shortened time requirements for various wild type and transgenic strains. Finally, we established the use of modified strains for experimental infections and underlined a host adaptation phase requirement.

**Conclusions/Significance:**

We devised an improved *T. congolense* model, which offers the opportunity to perform functional genomics analyses throughout the whole life cycle. It represents a very useful tool to understand pathogenesis mechanisms and to study potential therapeutic targets either *in vitro* or *in vivo* using a mouse model.

## Author Summary

*Trypanosoma congolense* is a parasite responsible for severe disease of African livestock. Its life cycle is complex and divided into two phases, one in the tsetse fly vector and one in the bloodstream of the mammalian host. Molecular tools for gene function analyses in parasitic organisms are essential. Previous studies described the possibility of completing the entire *T. congolense* life cycle *in vitro*. However, the model showed major flaws including the absence of stable long-term culture of the infectious bloodstream forms, a laborious time-consuming period to perform the cycle and a lack of genetic tools. We therefore aimed to develop a standardized model convenient for genetic engineering. We succeeded in producing long-term cultures of all the developmental stages on long-term, to define all the differentiation steps and to finally complete the whole cycle *in vitro*. This improved model offers the opportunity to conduct phenotype analyses of genetically modified strains throughout the *in vitro* cycle and also during experimental infections.

## Introduction

Animal African Trypanosomosis (AAT) is a severe disease caused by several species of African trypanosomes, flagellate protozoan parasites transmitted by an insect fly vector. It affects domestic livestock in sub-Saharan Africa and consequently has a severe economic impact [1]–[3]. AAT is widely spread affecting 40 countries situated in regions that could potentially be the most productive. The main pathological symptoms of animal trypanosomosis are weight loss, anaemia, and immunosuppression, but the mechanisms involved are poorly understood. It is estimated that 50 million cattle and 70 million small ruminants are at risk, costing the continent between 1.5 and 5 billion US dollars per annum. In terms of severity and consequences for productivity, *T. congolense* is the main causative agent of AAT. This parasite has a complex life cycle: bloodstream forms (BSF) proliferate in the blood of the infected mammalian host and are ingested by tsetse during the blood meal, procyclic forms (PCF) differentiate in the insect midgut, migrate to the proboscis (mouth parts) where they attach as epimastigote forms (EMF) and finally differentiate into infective metacyclic forms (MCF) that are transmitted to a new mammalian host during the next blood meal.

This parasite strongly hinders the agricultural development of the sub-Saharan region and an understanding of the virulence mechanisms is essential in order to block the parasite and/or the associated pathogenesis. Such studies require functional characterization of virulence genes based on genetic engineering. Genetic tools are well established in the *T. brucei* parasite with wide use of inducible transgene expression [4],[5]. More specifically, gene expression inactivation using RNA interference (RNAi) system greatly contributed to the functional analysis of *T. brucei* genes to such an extent that *T. brucei* has become the preferred model for trypanosomiasis studies [6],[7]. However, *T. congolense* is a strictly intravascular parasite whereas *T. brucei* can leave blood vessels and invade tissues implying differences in virulence mechanisms, host/pathogens relationships and pathogenic effects between the two species. Furthermore, while for *T. brucei* only the PCF and BSF stages can be cultivated *in vitro* and differentiation is limited to one step (from BSF to PCF), the whole life cycle of *T. congolense* can be reproduced *in vitro* (cultivation of all the developmental stages accomplishing all the differentiation steps) [8],[9]. Especially, the *in vitro* differentiation of the tsetse vector stages (from PCF to MCF) was described much earlier, this process is termed metacyclogenesis [10]. Therefore, it will be very informative and useful to use *T. congolense* not only as a model for AAT studies but also for deciphering the mechanisms underlying the different differentiation steps.

The major drawback we aim to overcome is that all cultivation techniques currently available are limited even for the metacyclogenesis process (which could take several months [10]) resulting in the quasi-absence of genetic tools. A single strain has been developed for the inducible RNAi system in PCF (TRUM183 strain) [11], and only one recent study described genetic modification of EMF (IL3000 strain) by transfection with a GFP vector and subsequent differentiation into MCF [12]. However, production of infectious BSF through differentiation of genetically modified parasites has never been achieved. In addition, there is an important heterogeneity between the *T. congolense* isolates used despite the inclination to refer to the IL3000 clone since the beginning of its genome sequencing project (Sanger Center, UK). And, there are disparities regarding the *in vitro* differentiation especially during metacyclogenesis and transfection efficiency [13] (our data). Finally, the question of the infectivity/virulence of wild type or genetically modified MCF differentiated *in vitro* has not been addressed until now.

Another severe restriction in this research field is the lack of stable long-term BSF culture. Some authors described BSF culture systems [14],[15] that turned out to be usable only for short-term applications eliminating the possibility of performing drug trials (requiring continuously dividing cultures) and above all, transfection assays, consequently resulting in the absence of functional genomics for *T. congolense* BSF.

Because the *T. congolense* model displayed significant limitations, we decided to undertake a systematic analysis of all the developmental stages regarding culture methods, differentiation efficiency, and transfection success rate by using 5 different wild type isolates and 2 genetically modified cell-lines. In this work we describe the success in developing an improved *in vitro* model and standardized culture methods.

## Materials and Methods

### Cell cultures

Primary cultures of bovine aortic endothelial cells (BAE) were routinely maintained in EGM2V medium (Lonza) in 24 well plates in a 5% CO_2_ humidified atmosphere at 37°C as described by the manufacturer. Cells were used between passages 9 and 20. Co-culture with parasites was conducted in the parasite medium.

### Trypanosome strains

Six *Trypanosoma congolense* strains of the Savannah type were used in this study. IL1180 originated from the Serengeti in Tanzania [16]. ILC-49 was originally isolated from a cow from the Transmara, Kenya, and was passaged in rodents [17]. This strain and its derivative, clone IL3000, were kindly provided by the International Livestock Research Institute, Nairobi, Kenya. STIB910 is a cloned derivative of STIB249 originally isolated in 1971 from a lion in Tanzania [16], and was kindly provided by R. Brun (Basel, Switzerland). TREU1457 was derived from a stock originally isolated in Nigeria (Zaria/67/LUMP/69) [18] kindly provided by C.A. Ross. TRUM183:29-13 is a genetically modified version of the TRUM183 strain, an uncloned serodeme, kindly provided by J.E. Donelson [11].

### Parasite culture

Details on culture media used are listed in Table 1. Procyclic forms (PCF) were routinely maintained in TcPCF-3 medium. Cells were maintained in log-phase culture at 27°C in 25 cm^2^ flasks by changing the medium every 3 days.

**Table 1:** Parasite culture media.

Code		Basal medium (mM)								Supplements (mM)							serum (% v/v)		
	Base powder	Hep	Gluc	Pyr	Ade	Hypo	Thy	Bac	Gln	Pro	2 Me	Cys	Aco	Cit	RBC	FCS	GS	FGS	SP
**TcPCF-1**	MEM	25				0.1			2	10			3	3		20			
**TcPCF-2**	MEM	25				0.1			2	10			3	3			20		
**TcPCF-3**	MEM	25				0.1			2	10						20			
**TcEMF-1**	MEM								2	10									
**TcEMF-2**	MEM								2	10						10			
**TcBSF-1**	MEM	25	5.5	1	0.04	0.1	0.02	0.02	2		0.2						20		5
**TcBSF-2**	MEM	25	5.5	1	0.04	0.1	0.02	0.02	2		0.2				0.5		20		5
**TcBSF-3**	MEM	25	5.5	1	0.04	0.1	0.02	0.02	2		0.2				0.5			20	5

MEM, Eagle's Minimum Essential Medium powder medium (M0643, Sigma-Aldrich); IMDM, Iscove's Modified Dulbecco's Medium (42200014, Invitrogen). Both media were supplemented with 2.2 g of sodium bicarbonate (Sigma-Aldrich). Basal medium components were resuspended in 1 L Versol water (Revol, Villeurbanne) and after adjustment of the pH to 7.3 with 5 N NaOH, the medium was filter sterilized (Millipore 0.22 um), stored at 4°C and used within 10 days. Before use, basal medium was supplemented with the components listed (Sigma-Aldrich) and serum. Hep, HEPES Acid; Gluc, D-Glucose; Pyr, Sodium Pyruvate; Ade, Adenosine; Hypo, Hypoxanthine; Thy, Thymidine; Bac, Bathocuproin; Gln, Glutamine; Pro, Proline; 2 Me, 2-mercaptoethanol; Cys, Cystein; Aco, Cis-aconitate; Cit, Citrate. RBC, Red Blood Cell lysate was obtained after centrifugation (1 min 2000 rpm) of 100 µl of mouse blood, and resuspending of the pellet in 50 µl sterile distilled water. FCS, Heat-inactivated (56°C, 30 min) fetal calf serum (Adgenix); GS, Goat Serum (Invitrogen); FGS, Fresh Goat Serum; SP, Serum Plus (SAFC Biosciences, United Kingdom).

Epimastigote adherent forms (EMF) appeared in this medium after several weeks of culture by maintaining the culture in stationary phase (change of half of the medium every 4 days). The entire culture supernatant was replaced with fresh medium every 2–3 days, after the colonies of EMF were well established. Alternatively, EMF could be cultivated in a few hours using a starvation medium named TcEMF-1. Briefly, 5 ml of PCF culture (≈10^7^ cells/ml) was collected by centrifugation (1600×g, 10 min), placed in the same volume of TcEMF-1 medium and incubated at 27°C in 25 cm^2^ flasks for 2 hours. Individual adherent EMF or colonies were observed after only 1 hour. Heat-inactivated fetal calf serum (10%, Adgenix) was added to the flask and this medium (named TcEMF-2) was used to maintain the obtained EMF culture as described above.

Metacyclic forms (MCF) developed in the EMF cultures after few days up to few weeks depending on the strain. MCF could be purified by DE52 anion-exchange column chromatography (Whatman Plc., Brentford, U.K.) [19]. Furthermore, the number of MCF produced per ml of EMF culture were quantified using a haemocytometer.

For bloodstream forms (BSF) cultures, endothelial cells were used as a feeder cell layer. BAE were seeded in 24-well plates. After one day, the medium was removed, cells were washed with PBS (phosphate-buffered saline: 137 mM NaCl, 10 mM Phosphate, 2.7 mM KCl, pH 7.4) and incubated in 1 ml of BSF specific medium. BSF were cultured in 2 different media, the TcBSF-3 was used during the adaptation phase from the infected mouse blood or from metacyclic differentiation and the TcBSF-1 was used for well-established cultures. BSF were always maintained at 34°C in a humidified atmosphere containing 5% (vol/vol) CO_2_.

### Cultivation of parasites isolated from mouse blood

Parasites collected from infected mouse blood could be either differentiated in PCF or cultivated as BSF. Cultures were initiated with tail blood of infected mice when parasitaemia reached a minimum of 10^8^ parasites/ml. 5 drops of tail blood were collected in 1 ml of TcPCF-1 or TCPCF-2 media for differentiation in PCF or in 1 ml of TcBSF-3 for BSF culture. A few derivatives of the TcBSF-3 medium were also used during the adaptation medium development phase by using horse, foal or lamb sera (Invitrogen) instead of goat serum. Centrifugation (200×g, 1 min) was used to remove the majority of red blood cells. Parasites present in the supernatants were subsequently incubated in 24 well plates at 27°C for PCF, and at 34°C in a humidified atmosphere containing 5% (vol/vol) CO_2_ for BSF.

For PCF, half of the medium was replaced as soon as the well-shaped parasite density in the upper part of the well reached the middle log phase (≈5.10^6^ cells/ml). This process could take a few days to a few weeks depending on the strain. Following this adaptation phase, half of the medium was changed every 2 days.

### Vectors

pLEW13, pLEW29 with a modified integration site (here named pLEW29c) and p2T7^Ti^/*αTUB* were kindly provided by J.E. Donelson [11]. pLEW20 is a typical expression vector for *T. brucei* [20], which allows inducible expression of proteins under the control of a strong pol I promoter regulated by two tetracycline operators. To facilitate the integration and the subsequent expression of reporter proteins in *T. congolense*, the integration site, the rRNA spacer region (1 to 121), was replaced by the βTUB region (1 to 134) of the pLEW13 vector. A PCR fragment from the pLEW13 was produced using specific primers containing ScaI and NdeI restriction sites respectively, sub-cloned in pGEM-T vector (Promega), and inserted in the pLEW20 ScaI-NdeI restricted plasmid. This new vector, named pLEW20c, was used to produce the enhanced green fluorescent protein (EGFP: optimized for expression and fluorescence in mammalian cells) or the Renilla luciferase (RLuc). PCR amplified fragments corresponding to EGFP and RLuc were inserted in the HindIII-BamHI restriction sites of the pLEW20c vector to produce pLEW20c-EGFP and pLEW20c-RLuc.

### Trypanosome transfection

Stably transfected strains were obtained with the Amaxa nucleofection method. The Amaxa Nucleofector® system (Lonza, Levallois-Perret, France) was used as described by the manufacturer. Briefly, a pellet of 10^7^ parasites was resuspended in 100 µl of Basic Parasite Nucleofector® solution, mixed with 15 µg of NotI linearized plasmid and subjected to nucleofection with a specific program. Amaxa solutions and programs used are listed in Table 2. Stably transfected trypanosomes were selected using BSF or PCF media supplemented with bleomycin for pLEW20c and p2T7^Ti^/*αTUB*, G418 for pLEW29c and hygromycin for pLEW13. Increasing concentrations of antibiotics were used for BSF selection (from 0.5 µg/ml to 2 µg/ml Bleomycin; 0.25 µg/ml to 1.25 µg/ml Hygromycin and 0.1 µg/ml to 0.5 µg/ml G418), while a fixed concentration was used for PCF (2 µg/ml Bleomycin, 2.5 µg/ml G418 and 6.25 µg/ml Hygromycin). During BSF selection, half of the medium was changed every two days by carefully removing it without disturbing the adherent parasites. For PCF, the medium was not changed until the parasite density reached at least 5.10^6^ cells/ml. After the selection phase, single clones were generated by limiting dilution, expanded and assessed by either PCR on genomic DNA (using primers specific of the vector amplifying the resistance gene) or luciferase activity quantification or GFP fluorescence depending on the vector used.

**Table 2:** Amaxa nucleofection assays.

Strain		Amaxa Solution	Program number	Vector	Positive assay/total	Transfectant analysis		
						PCR	Fluo	Activity
**PCF**								
	**IL3000**	T-cells	X-001, X-006, U-033	pLEW13	0/3			
		Parasitic 1	U-033	pLEW13	0/1			
		Parasitic 2	X-001	pLEW13	1/1	X		
			X-001	pLEW20c-GFP	1/1		X	
	**IL3000:13**	Parasitic 2	X-001	pLEW29c	1/2	X		
	**IL3000:13–29**	Parasitic 2	X-001	pLEW20c-GFP	1/1		X	
			X-001	pLEW20c-Rluc	1/1			X
			X-001	p2T7^Ti^/*αTUB*	1/2	X		
	**Treu183:13–29**	Parasitic 2	X-001	pLEW20c-GFP	1/1		X	
				pLEW20c-Rluc	1/1			X
				p2T7^Ti^/*αTUB*	1/1	X		
**BSF**								
	**IL3000**	T-cells	X-001, X-006	pLEW13	0/2			
		Parasitic 1	U-033	pLEW13	1/1	X		
		Parasitic 2	U-033	pLEW13	1/1	X		
			U-033	pLEW20c-Rluc	0/1			
	**IL3000:13**	Parasitic 2	X-001, U-033	pLEW29c	0/7			
	**STIB910**	T-cells	X-001, U-033	pLEW13	0/3			
			X-006	pLEW13	1/1	X		
		Parasitic 1	X-001, X-006	pLEW13	0/2			
		Parasitic 2	X-001	pLEW13	1/1	X		
			X-001	pLEW20c-Rluc	1/1			X
	**STIB910:13**	Parasitic 2	X-001, U-033	pLEW29c	0/8			
			X-001	p2T7^Ti^/*αTUB*	0/1			
	**IL3000:13–29**	Parasitic 2	X-001	pLEW20c-Rluc	1/1			X

Once selected, transfectants were analyzed by PCR on genomic DNA, observation of EGFP fluorescence, or Renilla luciferase activity measurement.

Transgene induction was achieved by growing the cells in the presence of 1 µg/ml of tetracycline.

### *In vitro* luciferase assay

The *Renilla* Luciferase Assay System (Promega, Charbonnières-les-bains, France) was used to measure *in vitro* luciferase activity as described by the manufacturer. Briefly, non-transformed and pLEW20c-Rluc-transfected *T. congolense* clones were grown to a total of 5.10^6^ parasites/ml and centrifuged (1500×g, 10 min). The pellet was washed with PBS, resuspended in 20 µl lysis buffer and added to 100 µl of the reaction mix. *Renilla* luciferase activity was monitored over 5 min every 10 sec after substrate addition by using an Optima microplate reader (BMG Labtech, Germany) and expressed as relative light units (RLU) per µg protein.

### Immunofluorescence analyses

PCF were collected by centrifugation (1600×g, 10 min) of culture supernatants. Individual EMF were collected by brief washing of the adherent cells with PBS and gentle scraping of the colonies with a cell scraper followed by centrifugation (1600×g, 10 min). MCF were purified from culture supernatants by DE52 anion-exchange column chromatography (Whatman Plc., Brentford, U.K.) [19]. BSF were collected by extensive flushing of the culture to remove adherent cells followed by centrifugation (1600×g, 10 min). To observe EMF colonies, glass coverslips were placed in 24-well plates and incubated with EMF until formation of colonies (the process can take a few days). To analyze BSF/BAE interactions, BAE cells were first incubated for at least six hours on glass coverslips placed in 24-well plates to allow them to adhere before addition of the BSF and co-culturing for several hours. Cells were fixed with formaldehyde as described [21]. Slides were then incubated with mouse anti-paraflagellar rod (PFR) antibodies (anti-PFR2 L8C4 monoclonal antibody diluted 1∶100), kindly provided by K. Gull, (Oxford, U.K.)) in PBS 0.1% (vol/vol) Triton X-100 0.1% (wt/vol), BSA for 30 min, washed three times with PBS and incubated for 30 min with Alexa Fluor 488 goat anti-mouse IgG secondary antibody (diluted 1∶100) (Molecular probes). Finally, cells were incubated for 5 min with 1 mg/ml 4′,6′-diamidino-2-phenylindle (DAPI) and mounted in Vectashield (Vector Laboratories). Cells were observed with a Zeiss UV microscope and images were captured using a MicroMax-1300Y/HS camera (Princeton Instruments) and Metaview software (Universal Imaging Corporation).

Direct observations of parasites in culture were also conducted: cells were observed with an Axiovert (Zeiss) UV microscope and images were captured by an Axiocan camera (Zeiss) and Axiovision 3.0 software (Zeiss).

### SDS-PAGE and immunoblotting

Total protein preparations of trypanosomes were obtained by lysis of live parasites with 2% (wt/v) SDS (10 µl per 10^6^ parasites) and heating at 100°C in the presence of a protease inhibitor cocktail (complete Mini, EDTA-free; Roche Diagnostics, GmbH). 10 µl of this lysate was loaded per well and separated by SDS-PAGE (12%) before transfer onto polyvinyl difluoride (PVDF) membranes (Immobilon-P, Millipore) and processed for Western blotting as described previously [22]. Monoclonal antibodies (mAb) #491 and #3C6 were kindly provided by T. W. Pearson and B. Loveless (Victoria, Canada). mAb #491 recognizes a carbohydrate epitope present on the protease-resistant surface molecule of *T. congolense* and on other surface molecules of *T. congolense* and *T. simiae* [12],[23]. mAb #3C6 was made by B. Loveless against recombinant CESP procured by N. Inoue (B. Loveless and T. W. Pearson, unpublished data). Membranes were incubated for 2 hours with dilutions of hybridome culture supernatant (1∶10) or mouse anti-*T. brucei* tubulin (1∶2,000). Antigen-antibody interactions were revealed with the Immobilon Western chemiluminescent horseradish peroxidase substrate (Millipore).

### Experimental infection of mice

Eight-week-old female Balb/c mice were purchased from Charles River Laboratories (L'arbresle Cedex France), NOD/SCID (NOD.Cg-Prkdescid Il2rgtm1 Wjl/Szj) were bred locally in specific pathogen-free conditions and used for experiments from five to six weeks of age. All animal studies adhered to protocols approved by the University of Bordeaux 2 animal care and use committee and the commission de genie genetique (Direction Generale de la Recherche et de l'Innovation). Balb/c mice either treated or not with the immunosuppressant cyclophosphamide (200 mg/kg, Sigma-Aldrich) or NOG mice were inoculated by intraperitoneal injection with variable amounts of infectious trypanosomes. Inoculated parasites were: (i) purified MCF, (ii) a mix of EMF and MCF from cell culture supernatants, (iii) BSF cultivated *in vitro* or from infected mouse blood. Parasitaemia was subsequently monitored daily by microscope observation. Blood samples were collected three times a week by tail bleed in 100 µl capillary tubes coated with Na-heparin. To determine the packed red blood cell volume percentage (PCV), capillary haematocrit tubes were sealed, centrifuged (15,000 rpm, 12 min) and analyzed with the haematocrit reader supplied by the manufacturer (Heraeus).

## Results

### Effect of medium composition on procyclic differentiation and culture

*T. congolense* PCF adaptation to an axenic culture system directly from infected mice blood was described previously [24]. However, with described methods and media, for most of the isolates, differentiation efficiency was low and required several months to attain stable culture. Furthermore, some strains could not undergo the adaptation process to axenic culture. Therefore, we aimed to standardize a simple method for rapid initiation from infected blood and subsequent stabilization of PCF in continuous culture.

Blood samples were collected from mice experimentally infected with various strains (IL3000, ILC-49, IL1180, TREU1457, TRUM183:13–29, STIB910). When the parasitaemia reached the mid log phase (∼5.10^6^ cells/ml), a few drops of tail blood were placed in 1 ml of media (TcPCF-1, TcPCF-2 or TcPCF-3; Table 1) and inoculated in 24-wells plates at 27°C after discarding most of the red blood cells by centrifugation. TcPCF media are derived from the 109-c medium [24] with variation in serum nature and percentage, and presence of cis-aconitate and citrate.

BSF were always transformed into PCF within 2 days but degenerated cells (abnormal shape, giant or clustered cells), unable to divide, appeared soon after and settled at the bottom of the well. These abnormal cells constituted the major part of the culture during the first 3–10 weeks before giving way to well-shaped dividing PCF. This adaptation phase is the critical step. When the culture reached at least 5.10^6^ cells/ml, the medium was changed with 1 ml of fresh medium. After a few days, the doubling time reached its maximum (8–15 hours depending on the strain) and the culture was transferred to 25 cm^2^ flasks and standard cultivation methods in TcPCF-3 medium were applied. The presence of 3 mM of cis-aconitate and citrate shortened the adaptation phase to at least 2 weeks, probably facilitating the differentiation process as described for *T. brucei* [25],[26]. Furthermore, this adaptation phase could only be conducted in TcPCF-2 (goat serum instead of fetal calf serum) for ILC-49 and TRUM183:13–29. In contrast, all the other tested strains preferred the TcPCF-1 medium. For the multiplication step, all strains grew faster in TcPCF-1. The length of time required to achieve a stabilized PCF culture in flask is reported in Table 3 for all the strains tested. We observed that even with this improved system, the time period required to achieve the process was strain dependant.

**Table 3:** *In vitro* cycle timing and virulence of various *T. congolense* strains.

Strain	Differenciation timing during metacyclogenesis (weeks)			Virulence of		Mice survival (Days)	Maximum parasitaemia (10^8^/ml)	Minimum PCV (%)
	Mouse>PCF	PCF>EMF	EMF>MCF	MCF	BSF			
**ILC49**	4 to 6	3 to 6	1 to 2	−	acute	3.9±0.3	1.8±0.2	44.7±1.5
**IL3000**	3 to 4	1 to 3	1 to 2	acute	acute	14.1±1.2	10.2±0.1	28.0±2.6
**IL1180**	3 to 4	3 to 6	1 to 2	chronic	chronic	48.7±4.6	5.4±0.3	25.3±3.0
**TREU 1457**	4 to 6	3 to 6	1 to 2	acute	acute	12.1±1.6	5.2±0.2	30.2±2.8
**STIB 910**	6 to 8	3 to 6	1 to 2	acute	acute	11.9±3.2	10.1±0.4	26.1±1.6
**TRUM183:13–29**	3 to 4	3 to 6	1 to 2	−	acute	15.2±4.3	5.6±0.3	31.3±2.1
**IL3000:13–29**	3 to 4	4 to 8	1 to 2	−	acute	13.2±2.6	5.4±0.1	28.6±1.4

Mice survival, Maximum parasitaemia and Minimum PCV were expressed as mean values calculated from experimental infections of at least 5 mice after infection with 10^7^ BSF obtained after at least 5 passages in mice. Timings were the same whether TRUM183:13–29 and IL3000:13–29 were transfected with pLEW20c derivatives or not. MCF were purified from *in vitro* cultured parasites by DE52 column chromatography, BSF were present in mouse blood after at least 5 passages.

### Procyclic transfection and genetic tool design

Transgene expression and RNA interference (RNAi) in *T. congolense* PCF have been reported previously [11],[27]. Specifically, the tetracycline inducible system first used in *T. brucei* (constitutive expression of RNA polymerase and tetracycline repressor, [5]) has been adapted for *T. congolense* by Inoue and colleagues in the TRUM183 uncloned serodeme [11]. Nevertheless, classical electroporation methods are not sufficient to ensure an acceptable success rate of transfection in several other strains (our data). For example, the most used and currently sequenced (Sanger Center) IL3000 strain is awkward for genetic manipulation (upon our data: less than 30% of electroporation assays lead to selection of stable transfectants). Because transfection efficiency could be greatly enhanced in the parasite *T. brucei* using the Amaxa nucleofection method [28], we applied this technology to *T. congolense*.

**Figure 1:**
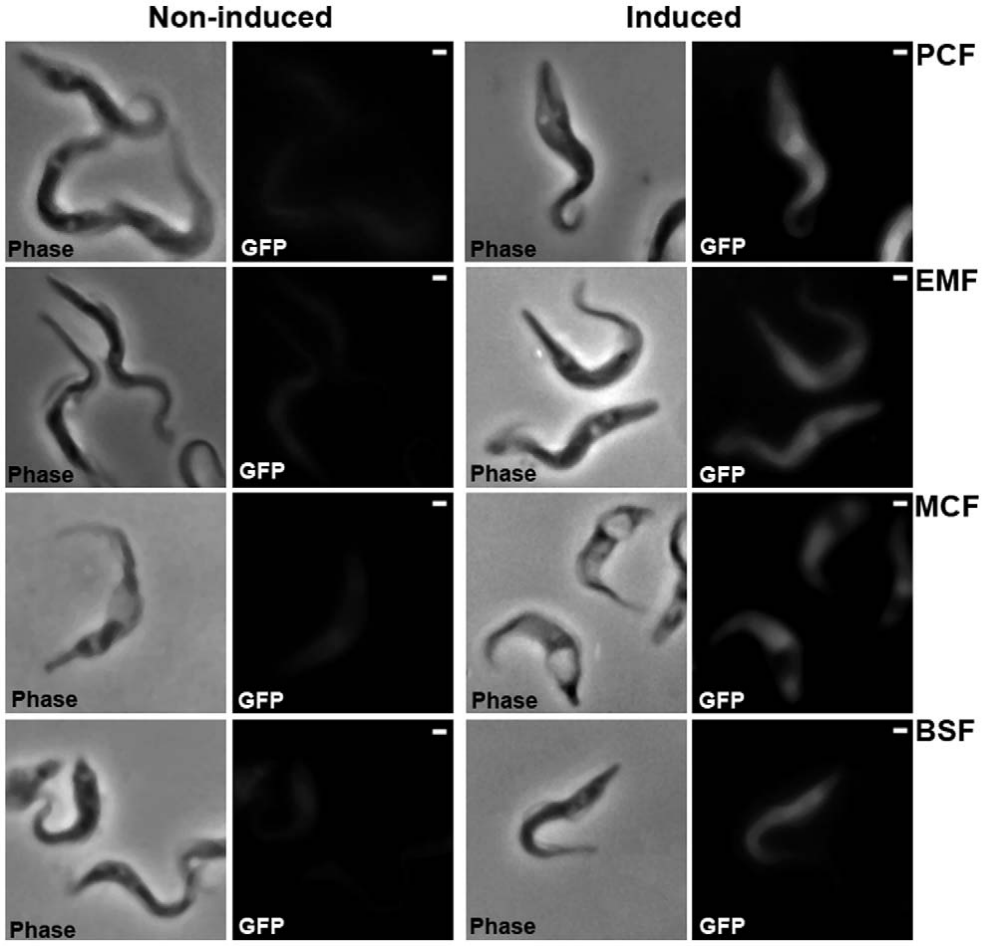
Inducible expression of EGFP through the *T. congolense* life cycle. Microscope images of the *in vitro* cultivated IL3000:13–29 strain transfected with pLEW20c-EGFP vector in all the developmental stages. Non induced and tetracycline (1 µg/ml for 48 h) induced cells were fixed and visualized in phase contrast and by EGFP fluorescence (images were recorded with the same exposure time). Scale bars = 1 µm.

**Figure 2:**
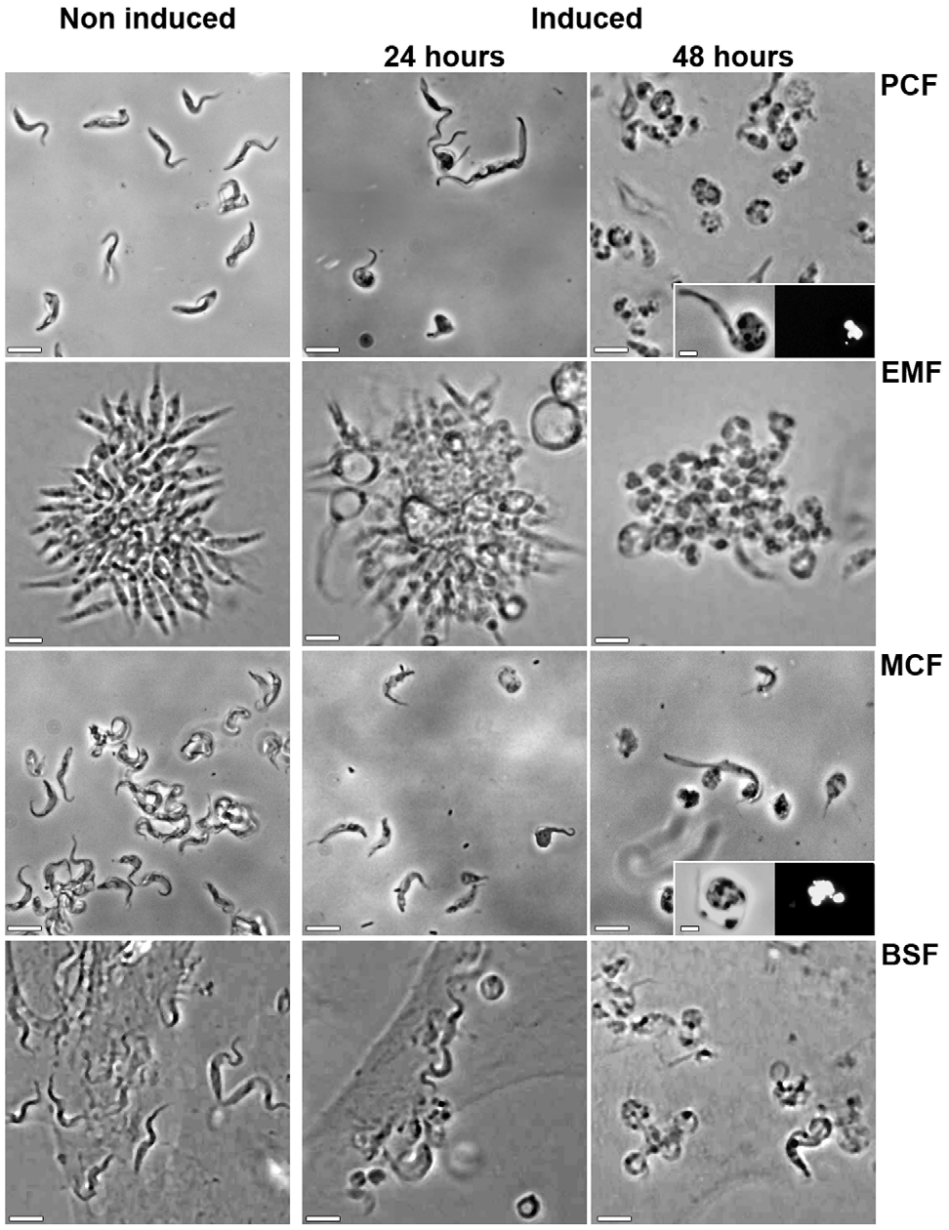
Inducible RNAi on tubulin genes through the *T. congolense* life cycle. Phase contrast microscope images of the *in vitro* cultivated IL3000:13–29 strain transfected with p2T7^Ti^/*αTUB* vector in all the developmental stages. Non induced and tetracycline (1 µg/ml for 24 h and 48 h) induced cells are presented. PCF, EMF colonies, MCF (after DE52 purification) and BSF on BAE feeder cell layer were observed directly in the culture medium. In the insets, PCF and MCF were fixed and stained with 4,6-diamino-2-phenylindole (DAPI) before observation in phase contrast. Scale bars = 10 µm.

To test this method on *T. congolense* PCF, we tried 3 different nucleofection programs and 3 different Amaxa solutions recommended by the manufacturer with different vectors as described in material and methods: p2T7^Ti^/*αTUB* as RNAi control experiment, pLEW20c-EGFP and pLEW20c-Rluc allowing inducible transgene expression of EGFP, Renilla luciferase, pLEW13 and pLEW29c inserted successively in the IL3000 genome to devise a tetracycline inducible system. The new cell-line was named IL3000:13–29. Stable transfection achievement has been assessed by PCR on genomic DNA and by GFP fluorescence or luciferase activity measurement. Results are reported in Table 2. The best results were obtained with the Parasitic 2 Amaxa solution and the X-001 program: independently of the *T. congolense* strain used, nearly 100% of the transfection assays led to selection of stable transfectants. With the other programs, electroporation led to cell death at much higher levels. The inducible operating system was also valued as shown in Figure 1, 2 and Table 4. Despite the absence of tetracycline, a basal expression level of GFP fluorescence and luciferase activity was observed; this is probably the result of a leakage of the tetracycline inducible system, which has already been described previously [20],[29]. Upon addition of tetracycline, transgene expression was enhanced as indicated by the rise in fluorescence intensity (Figure 1) and the 4-fold increase of luciferase activity (Table 4). For the RNAi experiment, as expected, the α-tubulin gene expression generated an altered cell morphology called the FAT phenotype [30] (Figure 2). Without tetracycline, this phenotype is absent, after tetracycline addition, the entire population displayed the lethal FAT phenotype within 48 hours. The absence of apparent leakage in this experiment is probably due to the natural enrichment of the culture with more tightly repressed cells due to the lethality associated with the FAT phenotype. This hypothesis is reinforced by prolonged selection time for p2T7^Ti^/*αTUB* transfectants and decrease of the doubling time in the course of the selection process.

**Table 4:** Luciferase activity in the *T. congolense* pLEW20c-Rluc transfected strains.

Strain	Clone	Tetra	Luciferase Activity (RLU/mg of proteins) in the different parasitic stages				
			PCF	EMF	MCF	BSFc	BSFb
Treu 183:13–29 pLEW20_LucR	C2	−	356±32	294±21	437±33	370±27	98±22
		+	1233±26	978±24	978±36	1061±32	
IL3000:13–29 pLEW20_LucR	C6	−	235±41	nd	nd	300±30	136±29
		+	1045±34	nd	nd	1288±19	
	B1	−				288±10	
		+				1300±35	
IL3000 pLEW20_LucR	B2	−				1467±32	

Tetra: absence (−) or presence (+) of tetracycline (1 µg/ml) in the culture. BSFc: cultivated BSF, BSFb: BSF from infected mice blood. nd: not determined.

### Metacyclogenesis of wild type and transgenic strains

Cultivation of all the life cycle stages from the tsetse vector has been described previously [8],[9]. However, using these protocols, production of infective MCF from insect form cultures could take few weeks to several months depending on individual strains of *T. congolense* [13]. The differences lay in cytoadherence timing, interval between attachment, infective MCF appearance and in the number of MCF produced. In order to shorten the metacyclogenesis process. We aimed to improve the differentiation efficiency in terms of timing and standardization.

**Figure 3:**
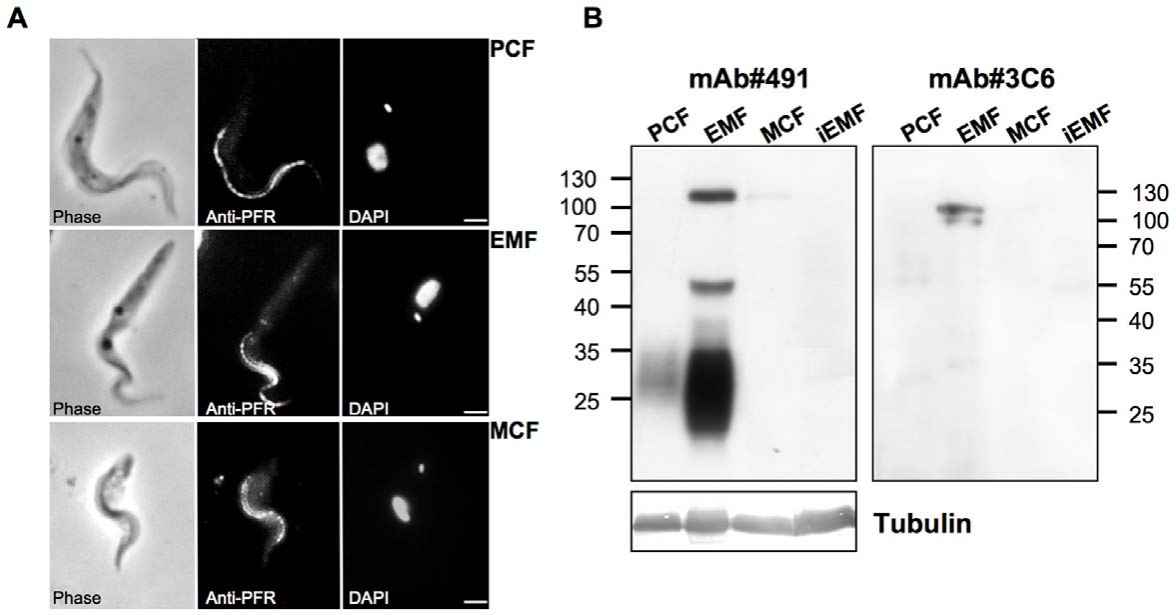
Morphological features and molecular markers analysis during metacyclogenesis. **A,** Wild-type individual IL 3000 cells were labeled with monoclonal anti-PFR antibody during metacyclogenesis. For orientation the fixed cells were also stained with 4,6-diamino-2-phenylindole (DAPI) and visualized in phase contrast. Scale bar = 1 µm. **B,** Western blot analysis of stage specific markers during the metacyclogenesis. 10^6^ cells of IL3000 were loaded per well. Tubulin was used as a loading control. Molecular weights are indicated on the blots side. iEMF: induced EMF in the absence of serum. Identical results were observed with the other *T. congolense* isolates and with transfected parasites.

First, to asses timing differences among strains, we tried to perform metacyclogenesis using the described methods with the wild type strains IL3000, ILC-49, IL1180, TREU1457, STIB 910 and the genetically modified cell lines TRUM183:13–29 and IL3000:13–29 transfected with either pLEW20c-Rluc, pLEW20c-EGFP or p2T7^Ti^/*αTUB*. PCF were inoculated with 5.10^6^ cells/ml in TcPCF-3 medium containing the optimal concentrations of glutamine and proline (2 mM glutamine and 10 mM proline) for metacyclogenesis as described by C. Ross [10]. These conditions were verified to be optimal (data not shown). After inoculation, half of the medium was changed every 2 days. To appreciate metacyclogenesis development, we considered several criteria to characterize the EMF and the MCF. (i) The first observed event was the EMF typical cytoadherence: parasites attached to the bottom of the flasks, grew and formed adherent bundles. (ii) Cell shape, size and kinetoplast and its attached flagella localization in relation to the nucleus change during metacyclogenesis process. These main morphological features were examined microscopically. For kinetoplast positioning, cells were labeled with a flagellar marker (anti-PFR antiserum) and stained with DAPI for orientation. (iii) MCF emergence was highlighted by purification on DE52 anion exchange chromatography. MCF infectivity assay in mice is described bellow. (iv) Stage specific markers were analyzed by western-blot, using two specific monoclonal antibodies. According to the literature, mAb #491 recognizes carbohydrate epitope shared by different surface molecules (such as PRS, GARP and CESP) differentially expressed during metacyclogenesis and mAb #3C6 binds to the EMF specific protein CESP [12],[23]. Results are presented in Figure 3 and Table S1. We confirmed that metacyclogenesis was successful as we chiefly observed the kinetoplast repositioning through the cycle (kinetoplast is always anterior to the nucleus in the EMF contrary to being on the posterior end in PCF and MCF) and MCF appearance within 1–2 weeks after cytoadherence. Furthermore, the expression pattern of stage specific markers is in agreement with published data on *in vitro* differentiated EMF: CESP is specifically induced in EMF stage and carbohydrate epitope presence is strongly increased during the PCF to EMF differentiation [12]. Nevertheless, we observed a large discrepancy among wild type strains with, for example, a required period of 3 to 10 weeks to observe cytoadherence for wild type strains. Moreover, differentiation was more difficult for genetically modified parasites since MCF getting required several months. For all the strains, the critical step seemed to be EMF differentiation.

Subsequently, we focused on the method to optimize this step by using the IL3000 strain. First, we attempted to influence the physiological state of the parasite by modifying the cell density of the culture. Unexpectedly, we observed a significant increase in the rate of cytoadherence (few days instead of weeks) when a density higher than 2.10^7^ cells/ml was used, corresponding to the stationary phase. All the strains were subjected to this treatment and EMF differentiation followed by MCF differentiation was noted (results are reported in Table 3). With this treatment, all the strains were able to pass through metacyclogenesis and parasites fit all the stage specific features described above, but variations in EMF differentiation timing were observed. Conversely MCF always appeared within 2 weeks. One of the features of stationary phase is depletion of metabolites in the medium. Taking this into consideration, we used a medium containing only base powder, glutamine, proline and no serum, TcEMF-1. Surprisingly, in this medium, individual cells and bundles of adherent IL3000 cells appeared after 30 minutes and reached a maximum after 2 hours. Prolonged exposure time in the absence of serum resulted in cell death. Therefore, after 1–2 hours, we added 10% FCS in this medium to produce TcEMF-2. Half of the medium was then changed every two days and it was found that MCF always appeared after 1–2 weeks. Adherent cells induced by serum depletion (named iEMF for induced EMF) displayed the main EMF criteria (cytoadherence, shape, kinetoplast positioning and ability to differentiate in MCF) but exhibited differences in molecular markers (Figure 3B). Indeed, no signal was observed either with mAb #491 or #3C6, which implies that CESP is not expressed and the carbohydrate epitope is absent.

This rapid method was tested on all the strains and worked even for transgenic strains: iEMF always appeared within 1 hour and MCF within 1–2 weeks. To confirm the potential use of the process to conduct functional analysis through the life cycle, GFP fluorescence, luciferase activity and FAT phenotype were determined in transfected strains either induced or non induced (Figure 1, 2 and Table 4). As expected, the results are similar to those of PCF. This process provides the opportunity to test phenotypes rapidly and efficiently throughout the parasite's developmental stages of the tsetse fly.

### Impact of *in vitro* culture on infectivity and virulence

To assess infectivity and virulence of *in vitro* MCF, we injected 10^7^ parasites into Balb-c mice and measured the level of parasitaemia. The different strains were not equally infective for mice (Table 3). IL3000, TREU1457 and STIB910 produced an acute parasitaemia with a single peak, a high parasite load, a strong anaemia and death within 2 weeks (Figure S1). IL1180 resulted in a chronic infection with a fluctuating blood parasitaemia leading to a survival time greater than one month (Figure S1). As for ILC-49 and transgenic cell lines, MCF were not infective and consequently no parasites were observed in the blood.

**Figure 4:**
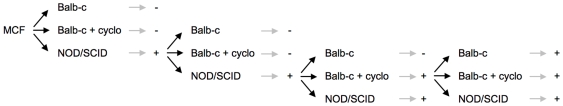
Infectivity of TRUM183:13–29 cell-line through mice passages. 10^7^ *in vitro* differentiated MCF or a volume corresponding to 10^7^ parasites of infected mice blood were injected in Balb-c, Balb-c pretreated with cyclophosphamide or NOD/SCID mice. Development of parasitaemia was monitored by microscopic observation of mice blood every two days during 2 months. − means that no parasite could be observed during the time period of observation, + means that parasitaemia developed during the time period of observation.

Previous parasitological studies associated *T. brucei* virulence differences with the capacity of sub-cellular membrane fractions to induce immunosuppression [31]. For this reason, we used immunosuppressed mice (cyclophosphamide pretreated Balb-c) and immunodeficient mice (NOD/SCID) in order to by-pass the lack of infectivity and allow these strains to develop in the murine host (Figure 4). MCF resulted in parasitaemia only in immunodeficient mice with a long prepatent period (at least 1 month). On the other hand, once parasites appeared, we observed a high parasite load, development of anaemia and death within two weeks. Subsequently, 10^7^ BSF containing blood was injected into new Balb-c mice pretreated or not with cyclophosphamide or into NOD/SCID mice. We obtained similar results as the parasitaemia only developed in NOD/SCID mice albeit with a shorter prepatent period (after 2 weeks). During the second passage, BSF were observed not only in NOD/SCID mice but also in immunosuppressed Balb-c mice. Following the third passage, blood samples were infective in immunocompetent Balb-c mice. For the fourth passage we observed the same infectivity and virulence through passages resulting in the development of acute parasitaemia with parameters comparable to those obtained with blood BSF stocks never cultivated *in vitro* (field isolates directly adapted to rodents) (Table 3).

To gain a better understanding of this phenomenon, we examined the possibility of a requirement of a host adaptation step for these non-infective MCF. Indeed, it has been described that MCF of the same *T. congolense* clone produced by tsetse fed with various host blood displayed virulence differences in mice and required an adaptation period to develop in mice [32]. Furthermore some *T. vivax* field isolates have been adapted to rodents using intraperitoneal inoculation of goat serum [33]. We therefore, injected 500 µl of goat serum intraperitoneally in immunosuppressed Balb-c mice one day before parasite infection and every subsequent day until BSF were observed in the blood. We observed parasites as soon as one week after MCF inoculation and mice eventually developed an acute parasitaemia. This protocol did not work with immunocompetent mice. The same treatment was required for the first passage in mice. From the second passage, goat serum injection was not essential and blood samples were infective in immunocompetent Balb-c mice. These data confirmed the existence in some strains of an adaptation process to the murine host.

### Setting-up long-term BSF cultures

We aimed to develop long-term cultures of *T. congolense* BSF in order to conduct drug trials and genetic modifications. We followed the described procedures to obtain BSF from infected mice blood [14]. Dividing BSF were easily obtained but parasites soon began to degenerate and the culture collapsed after 3 to 10 days depending on the strain. We investigated the consequences of varying media components such as serum and base powder. We inoculated standard *T. brucei* BSF medium containing either IMDM or MEM base powder and derived media containing 20% of various sera (horse, foal, lamb, goat) as described earlier [34]. The results showed a preference for goat serum and MEM base powder (data not shown). Supplementation of these media with “serum plus” increased cell density and attachment (TcBSF-1). We also examined temperature effect by cultivating parasites between 34°C and 37°C and observed higher longevity at 34°C. But despite these improvements, the culture conditions were not sufficient for long-term culture.

To go one step further with the culture system, we used endothelial cells as feeder cell layer. Indeed, strong interactions between vascular endothelium have been demonstrated *in vivo* [35],[36] and *in vitro* with BAE [37],[38]. Addition of BAE greatly improved the BSF condition as cells promptly interacted with BAE, proliferated faster and could be cultivated for 3–4 weeks. Nevertheless, this was not sufficient for the selection process of stable transfectants since some major drawbacks remained: cultures were very sensitive and preservation through freezing was not possible. Eventually, we managed to settle BSF adaptation by adding RBC lysate to the culture (TcBSF-2), and by using fresh goat serum provided directly after sampling thus avoiding alteration through preservation or treatment (chemical or irradiation) of the commercial serum (TcBSF-3). Therefore, the adaptation process had to be carried out in TcBSF-3, this phase lasted few weeks before stabilization of the culture. All the strains tested could be cultivated for at least a month but stabilization has been achieved only for IL3000, IL3000:13–29 and STIB 910. When the culture is stable, commercial goat serum can be used and RBC lysate and serum plus were no longer essential to sustain growth.

**Figure 5:**
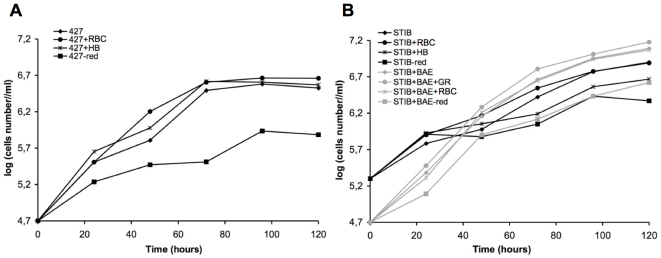
Comparison of *T. brucei* and *T. congolense in vitro* growth. **A,** *T. brucei* 427 BSF have been inoculated at 5.10^4^ cell/ml in TcBSF-1 (Table 1) medium at 37°C in a humidified atmosphere containing 5% (vol/vol) CO_2_. **B,** *T. congolense* STIB910 BSF have been inoculated at 5.10^4^ in presence of BAE (black curves) or at 2.10^5^ cell/ml in absence of BAE (grey curves) in TcBSF-2 (Table 1) medium at 34°C in a humidified atmosphere containing 5% (vol/vol) CO_2_. Parasites were cultured in their specific medium for five days (——⧫) or in medium supplemented with red blood cell lysate (resuspension of red blood cells vol/vol in cold water and 100 fold diluted in medium) (——•), or with hemoglobin (100 µg/ml) (—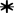), or in absence of reducing agents (Bathocuproin, 2-mercaptoethanol and cystein were removed) (——▪). Cells were counted every 24 h.

To test our BSF culture system, we compared growth of *T. brucei* and *T. congolense* in different media (Figure 5, Table S2). RBC lysate and haemoglobin slightly improved growth rates for both species and the absence of reducing agents affected *T. brucei* to a greater extent than *T. congolense*. The absence of BAE in *T. congolense* culture had a great impact on growth rate, and furthermore, the culture was more sensitive as it was not possible to inoculate with less than 2.10^5^ cells/ml. It was also observed that in the presence of BAE *T. congolense* reached the stationary phase later and supported a higher cell density than *T. brucei* (2.10^7^ cells/ml vs. 4.10^6^ cells/ml).

In addition, we subjected *T. congolense* MCF to differentiation with this adaptation system in order to directly obtain BSF *in vitro* avoiding the murine step. We obtained similar results: all the strains differentiated in BSF and were cultivated for one month and we completely stabilized IL3000, IL3000:13–29 and STIB910 for long-term culture. Infectivity and virulence of *in vitro* cultured BSF were tested by inoculating mice with parasites cultured for several months. Identical results were obtained as for MCF (Table 3). To complete the *in vitro* cycle, the cultivated BSF were subjected to PCF differentiation as described earlier. All the strains tested passed through this step and gave rise to a stable PCF culture.

Finally, we cultivated transfectant strains and managed to observe the corresponding phenotype: GFP fluorescence, luciferase activity and FAT phenotype (Figure1, 2 and Table 4). This experiment proved unambiguously the ability to fulfill phenotype analysis of genetically modified cell lines during the complete life cycle of *T. congolense*.

### Transfection experiments on *T. congolense* bloodstream forms

In the same way as PCF, we used the Amaxa nucleofection technology to transfect BSF. It turned out that BSF transfection was very difficult. Results are summarized in Table 2. We tried to construct the inducible system based on the tetracycline repressor directly in BSF and managed to achieve the first step, which was the integration of the pLEW13 vector into the genome. However, we failed to insert the second vector, pLEW29c, despite numerous endeavors. We then attempted to perform a constitutive RNAi experiment or reporter gene expression. We obtained successful results with the pLEW20-Rluc vector and highlighted luciferase activity (Table 4). We also achieved inducible expression of luciferase by directly transfecting the IL3000:13–29 cell line produced from the *in vitro cycle*. Direct transfection of *T. congolense* BSF is therefore possible but remains arduous as it took several weeks and the success rate was low.

## Discussion

**Figure 6:**
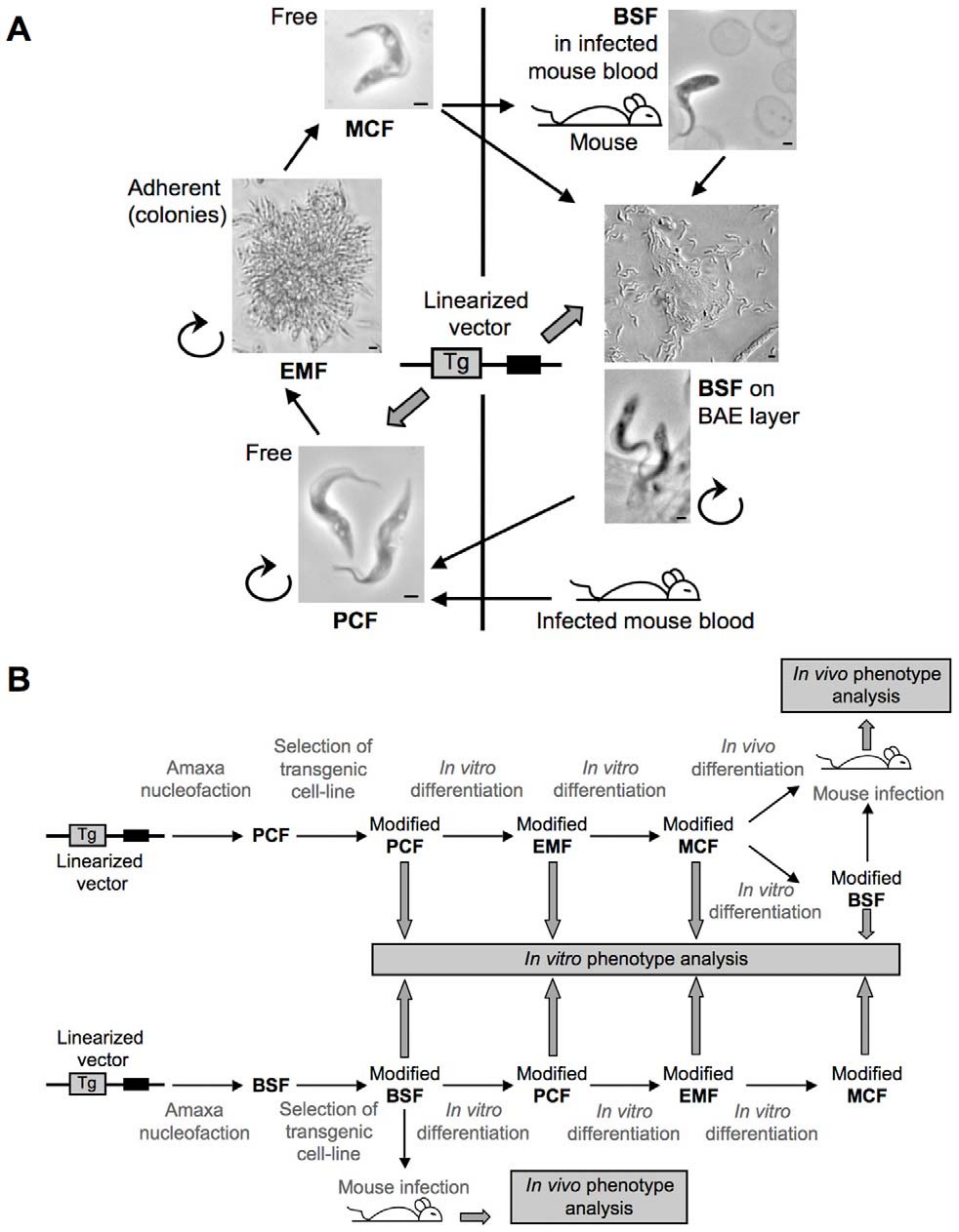
*In vitro* culture system and genetic tools for *T. congolense*. **A,** The different developmental stages cultured *in vitro* are represented as individual cells for PCF and MCF as adherent cells forming colonies for EMF and on BAE layer for BSF. Scale bar = 1 mm for PCF, MCF, BSF in infected mouse blood and the lower photo of BSF on BAE layer. PCF, EMF and BSF are dividing cells as represented by the rounded arrow. BSF differentiation can be achieved either by infection of mice and culture from blood or directly *in vitro* on BAE layer. **B,** Scheme of transgenic cell-line analysis through the cycle starting from PCF (top) or BSF (bottom) transfection. Linearized vectors used in transfection assays are represented by a line and two boxes, the black one represents the selection marker and the grey one represents the transgene (Tg).

The development of a continuous culture system represents a major breakthrough in *T. congolense* research. Indeed, parasite biology studies imply understanding of its complex life cycle including differentiation, environment adaptation, signaling, virulence and pathogenesis, which become accessible once powerful genetic tools such as RNAi are exploitable throughout the life cycle. For the first time, we have conducted a systematic study, which allowed us to define the best *in vitro* culture conditions for each stage in terms of differentiation efficiency followed by long-term *in vitro* culture design. In this way, we succeeded in reproducing the complete *in vitro* life cycle allowing the study of genetically modified cell-line phenotypes throughout the cycle (Figure 6).

Firstly, we designed a new enriched adaptation medium which enabled the isolation of BSF directly from the blood of infected mice and their long-term *in vitro* culture, which makes them amenable to *in vitro* drug trials, preservation through freezing and transfection assays. However, transfection of BSF remains a difficult task as we were unable to perform direct RNAi experiments but did succeed in overexpressing the luciferase reporter gene. Secondly, we improved the efficiency of PCF transfections and set up a tetracycline inducible system for the *T. congolense* reference strain (IL3000, the strain used by the Sanger Center for the genome sequencing project). We validated this system by the inducible expression of reporter genes (GFP and Luciferase) and an inducible RNAi experiment targeting the essential tubulin gene as a control. Thirdly, we standardized metacyclogenesis techniques, not only on wild type isolates, but also on genetically modified parasites (either for transgene expression or RNAi) and the time needed to achieve the differentiation has been dramatically shortened to ∼10 days instead of months. Finally, BSF differentiation could either be achieved by infecting mice or directly *in vitro*. Then, PCF transfection followed by metacyclogenesis became an efficient, rapid and reliable way to study gene function in all the developmental stages as well as during the differentiation steps. For the first time, powerful tools such as reverse genetics (gene expression inactivation and characterization of resulting phenotypes), becomes now available for this devastating parasite.

Adaptation of trypanosomatids to rodents for experimental infections was essential to analyze the host-pathogen interaction [39]. Specifically the use of inbred mouse models have proven to be a valuable tool in pathology research [40]. Distinctness in mouse susceptibility to trypanosomiasis among the mouse strains is always observed. For example, for *T. congolense* and other trypanosome species, BALB/c mice (compared to C57Bl/6) are the most susceptible [41]–[43]. Discrepancy in virulence among *T. congolense* isolates has also been reported in ruminant livestock as well as in laboratory animals [44],[45]. To go one step further in the understanding of virulence mechanisms, it is essential to analyze the phenotype of genetically modified parasites during experimental infection. This is the reason why we assessed the impact of long-term *in vitro* culture and of minor genome modification on parasite infectivity and virulence. Results showed that BALB/c mice are less susceptible to transfected cell-lines as well as to 1 of 5 tested wild type savannah strains after a complete *in vitro* life cycle. This deficiency in infectivity can be overcome by using immunodeficient animals or goat serum injected immunosupresssed mice. Furthermore, infectivity levels return to normal after few passages in the murine host. The survival time of infected mice is always correlated with the parasitaemia peak arising and since the parasites developed, we concluded that disease parameters like anemia are not influenced by *in vitro* passage. These data indicate that while in some cases the ability of the parasite to multiply and be maintained in the host is lowered, parasite virulence is not affected. We propose that infectivity decrease relies on a host adaptation step. Development of parasitaemia in the mammalian host implies for the parasite to fit the new environment in terms of energy supplying and trypanocides conditions. Impairment of the immune system could allow a time period sufficient for the parasite to adapt to its new physiological state. Goat serum injections might provide the parasite with essential factors like nutriments and create a microenvironment promoting the emergence of host adapted parasites that are able to develop in mice blood. Such phenomenon has been suggested for *T. vivax* adaptation to laboratory rodents using multiple passages in irradiated rats and intraperitoneal injection of goat serum [33].

This idea is in harmony with the requirement of goat serum supplemented medium for *T. congolense* BSF *in vitro* culture. Composition of this serum must suit the specific needs for parasite growth. The concentrations of vitamins and related metabolites vary from one sera to another. Goat serum is one of the most commonly used to enhance cell multiplication [46],[47]. The greatest efficiency of fresh goat serum (not treated) reinforced the hypothesis that the presence of labile components is required for the parasite well-being. One can also notice that the 3 strains impaired for infectivity (ILC-49, TRUM183:13–29 and IL3000:13–29) are those which need goat serum to promote their *in vitro* differentiation in PCF. These findings illustrated that even for closely related strains (all are of the Savannah genetic group), subtle differences in physiological requirements might exist.

Another important factor to promote BSF *in vitro* growth is the presence of the erythrocyte lysate, which became dispensable after some time. Haemoglobin is a component of red blood cells and we showed that its presence in culture medium enhances the growth rate of the parasite cultures. The first culture media described for *Trypanosoma spp*. were always supplemented with blood lysate, then replaced by hemin [48]. However, neither haemoglobin nor hemin, are sufficient to ensure BSF adaptation from infected mice blood (data not shown). Hemin is also essential for trypanosomatids PCF differentiation and growth [24],[49]. The parasite's inability to synthesize heme explains this requirement, since heme containing proteins like cytochrome c [50], are essential for parasite viability. However, erythrocyte lysate might provide the parasite with other unknown components, which are essential during the adaptation phase.

Generally, *T. congolense* culture has to mimic the host environment. Thus, a high amino acid concentration in insect forms media reflects the high proline content in tsetse hemolymph subsequently used for energy metabolism of the parasite. An even more striking similarity is the use of BAE layer to cultivate BSF. Indeed, in infected animals, *T. congolense* BSF are found adhering to erythrocytes and to endothelial cells of the microvasculature by their flagellum [35],[36],[51]. This requirement is no longer essential after an adaptation phase. Finally, production of infective MCF requires strong adherence of EMF mimicking the bundles formed in the fly proboscis. However, parasite journey in fly midgut then in proboscis is complex and can't be fully reproduced *in vitro*. Especially coat antigens expression is refined and follows a defined kinetic in the different insect stages. For example, one of the major coat protein, GARP, is weakly expressed in early PCF, present in the midgut then downregulated before being strongly expressed in EMF isolated from proboscis [23]. In culture, this marker is expressed in both stages PCF and EMF, reflecting an artefactual absence of regulation during *in vitro* EMF differentiation [23]. Furthermore, Butikofer *et al.* [23] did not find reactivity with mAb #491 on EMF from tsetse, although Sakurai *et al.* [12] did find increased reactivity with EMF compared to PCF within *in vitro* culture. Similarly, the iEMF phenotype (absence of CESP expression and of the carbohydrate marker) may represent another illustration of the importance of the environmental factors on differentiation. Indeed, the difference observed in surface markers may result from the absence of serum at the time of differentiation or from the stress induced by the sudden environment change. Nevertheless, despite the absence of those markers iEMF are able to differentiate into infective MCF suggesting that those surface markers expression is not essential for *in vitro* EMF differentiation but must be involved in the *in vivo* process where parasites have to attach themselves to the wall of the labrum in the food canal to transform into EMF. The iEMF stage could also represent a transitory stage in the insect. Furthermore the absence of CESP expression does not hinder *in vitro* iEMF adhesion implying that this protein is not essential for cytoadherence and that other proteins might be involved. Successful culture and differentiation of iEMF and EMF combined with targeted gene deletion through RNAi represent powerful tools to understand the elaborated mechanism of the metacyclogenesis process encountered in the insect.

Parasites display a great ability to adapt but the required time period for this adaptation is the limiting step in the initiation of stable culture of the different developmental stages as well as for experimental infections. We succeeded to go through this limiting step to secure cultures of all the *T. congolense* developmental stages. Standardization of media and methods using various strains, more or less demanding, allowed the design of the complete *in vitro* lifecycle of all the tested strains. Definition of these optimal conditions also greatly improved the success rate of PCF nucleofection, that should be very useful for routine genetics based analysis.

To conclude, the ability to dominate the *in vitro* metacyclogenesis in combination with the transgenic PCF technique provides an essential tool to investigate the functional role of *T. congolense* genes throughout the cycle as well as *in vivo* during experimental infections in mice.

## Supporting Information

Figure S1 Acute and chronic infection in mice. 5 Balb-c mice were infected with 104 parasites of IL3000 or IL1180 *T. congolense* strains. Parasitaemia and PCV were measured and expressed as mean values.
(0.29 MB TIF)

Table S1 Metacyclogenesis timing of various *T. congolense* strains. Cytoadherence timing indicates the time required to observe an increasing number of adherent cells at the bottom of the flask. MCF number (mean of two experiments) was determined by counting parasites after a DE52 chromatography, the experiment was done on 10 ml culture in Tco-PCF3.
(0.20 MB TIF)

Table S2 *T. brucei* and *T. congolense* growth rates in different media. Presence (+) or absence (−) of BAE feeder cell layer. Normal, corresponds to routinely used medium for each species in vitro culture. RBC, medium was supplemented with red blood cell lysate as previously described. HB, medium was supplemented with haemoglobin (100 mg/ml). red, reducing agents of the medium (2-mercaptoethanol, bathocuproin and cystein) were removed.
(0.09 MB TIF)
