# Supplementary figures and images for: Complete In Vitro Life Cycle of Trypanosoma congolense: Development of Genetic Tools
Source: PLoS Negl Trop Dis. 2010 Mar 2;4(3):e618. doi: 10.1371/journal.pntd.0000618 (PMC2830455; doi:10.1371/journal.pntd.0000618)

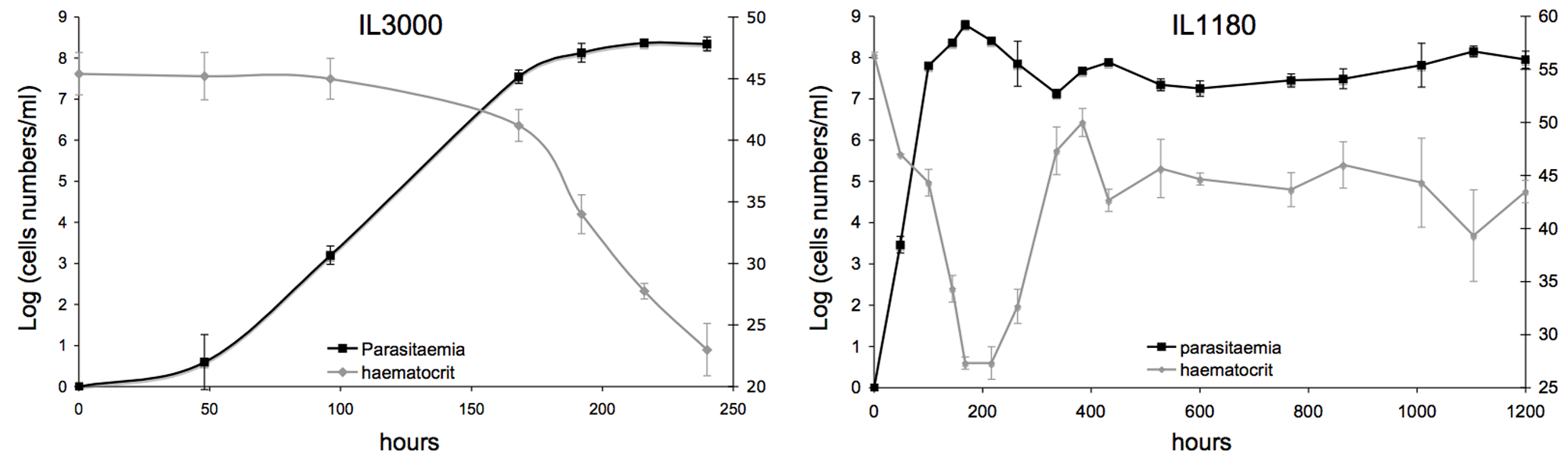

Supplement: Figure S1 — Acute and chronic infection in mice. 5 Balb-c mice were infected with 104 parasites of IL3000 or IL1180 T. congolense strains. Parasitaemia and PCV were measured and expressed as mean values. (0.29 MB TIF) [file pntd.0000618.s001.tif]

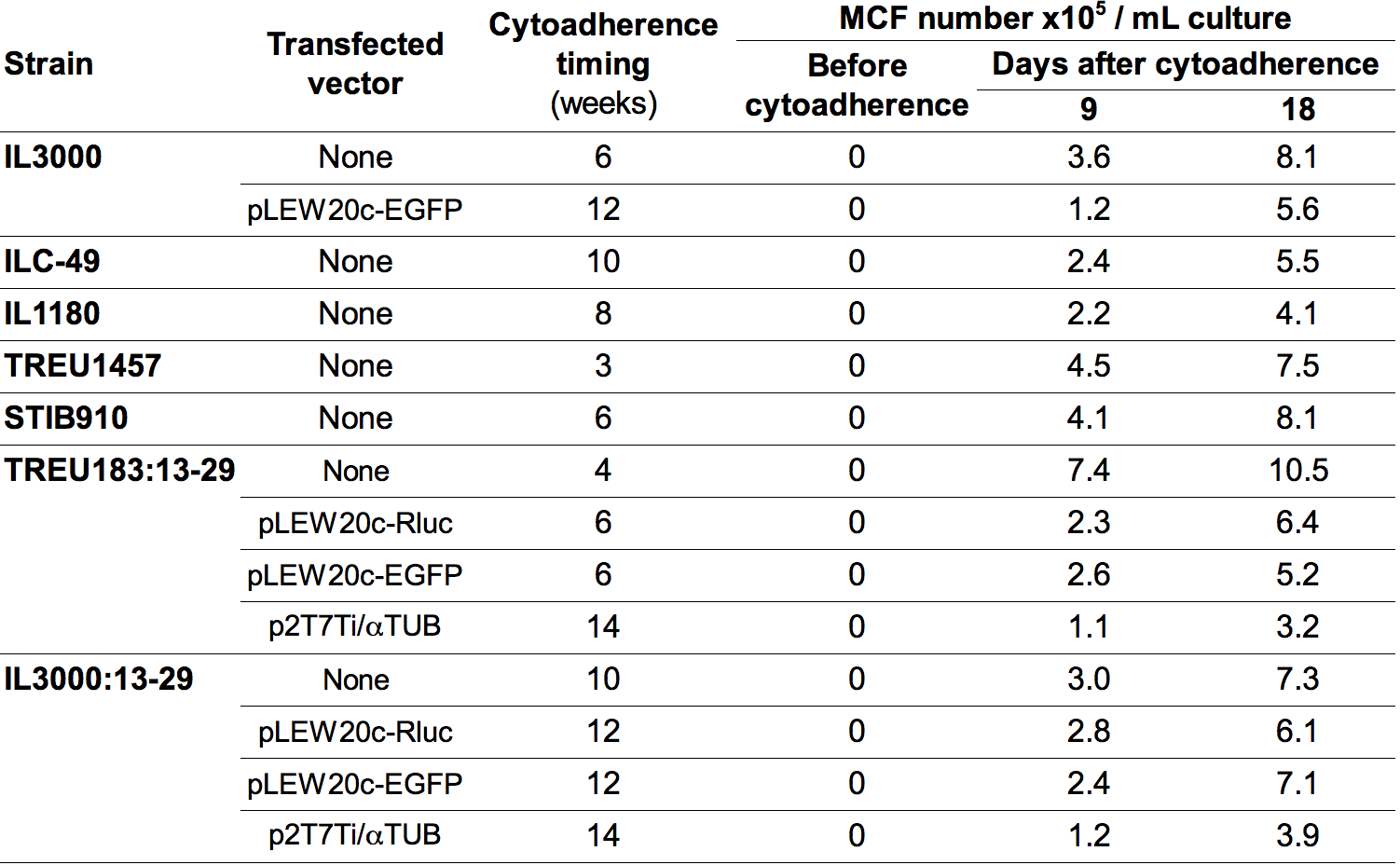

Supplement: Table S1 — Metacyclogenesis timing of various T. congolense strains. Cytoadherence timing indicates the time required to observe an increasing number of adherent cells at the bottom of the flask. MCF number (mean of two experiments) was determined by counting parasites after a DE52 chromatography, the experiment was done on 10 ml culture in Tco-PCF3. (0.20 MB TIF) [file pntd.0000618.s002.tif]

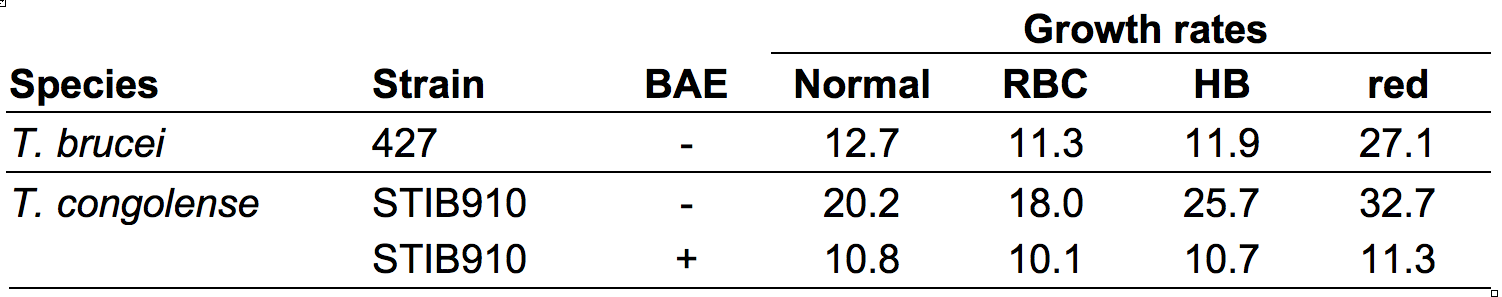

Supplement: Table S2 — T. brucei and T. congolense growth rates in different media. Presence (+) or absence (−) of BAE feeder cell layer. Normal, corresponds to routinely used medium for each species in vitro culture. RBC, medium was supplemented with red blood cell lysate as previously described. HB, medium was supplemented with haemoglobin (100 mg/ml). red, reducing agents of the medium (2-mercaptoethanol, bathocuproin and cystein) were removed. (0.09 MB TIF) [file pntd.0000618.s003.tif]
